# High-flow oxygen therapy for extubation failure prevention in high-risk critically ill patients: a randomized multicenter trial

**DOI:** 10.1186/2197-425X-3-S1-A164

**Published:** 2015-10-01

**Authors:** R Fernandez, C Subira, F Frutos, G Rialp, C Laborda, JR Masclans, G Hernandez

**Affiliations:** Hospital Sant Joan de Deu / Fundacio Althaia, Manresa, Spain; Universitat Internacional de Catalunya. CIBERES, Barcelona, Spain; H. Universitario de Getafe, Madrid, Spain; H. Son Llatzer, Mallorca, Spain; H. U. Valle Hebron, Barcelona, Spain; H. Virgen de la Salud, Toledo, Spain

## Introduction

Extubation failure in critically ill patients has associated morbidity, but it cannot be safely predicted or avoided. Preventive NIMV has proved beneficial only in hypercapnic patients. We hypothesized that High-flow oxygen therapy may reduce postextubation respiratory failure due to gas humidification, avoidance of lung collapse by moderate PEEP, and work of breathing reduction by dead-space washing.

## Objectives

Our objective was to reduce postextubation respiratory failure with High-flow in high-risk patients.

## Methods

Randomized multicenter trial in patients who successfully passed a spontaneous breathing trial. Only patients with criteria for high-risk of failure were randomized to receive conventional oxygen or High-flow oxygen (Optiflow®: Fisher Paykel) for 24 hours post-extubation. Primary outcome was respiratory failure within 72 hours postextubation. Secondary outcome were: reintubation, ICU and hospital length of stay, and survival. Statistical analysis included multiple logistic regression models.

## Results

The study was stopped after 18 months due to low recruitment. We enrolled 155 patients: 77 with conventional and 78 with High-flow oxygen. Groups were very similar at enrollment. All patients tolerated the High-flow system. We found the projected reduction in postextubation respiratory failure (20.5% vs. 27.3%, p = 0.3) that failed to reach statistical significance due to sample size. Similarly, the benefit in reintubation (16.7% vs. 19.5%, p = 0.6) didn´t reach significance. We found no differences in ICU length of stay, hospital length of stay, and survival. By a logistic regression model, we were able to independently associate high-flow with reduction in postextubation respiratory failure (OR 0.4 p = 0.049), by including COPD, cancer, length of MV, obesity, and cardiac failure in the model.

## Conclusions

High-flow oxygen therapy for 24-h may reduce postextubation respiratory failure in high-risk critically ill patients.

